# Validating the efficacy of interval appendectomy for acute appendicitis: representative three cases with different etiologies

**DOI:** 10.1186/s40792-020-00971-1

**Published:** 2020-08-12

**Authors:** Yuta Kasagi, Keita Natsugoe, Takehiko Aoyagi, Yoshinari Nobutou, Eiji Tsujita, Mayumi Ishida, Sosei Kuma, Katsumi Takizawa, Hideaki Uchiyama

**Affiliations:** 1 Department of Surgery, National Hospital Organization Fukuoka Higashi Medical Center, Koga, 811-3113 Japan; 2 Department of Pathology, National Hospital Organization Fukuoka Higashi Medical Center, Koga, 811-3113 Japan

**Keywords:** Interval appendectomy, Malrotation, Ulcerative colitis, Appendiceal carcinoma

## Abstract

**Background:**

Appendectomy for acute appendicitis (AA) is considered one of the most common emergency surgeries. However, emergency appendectomy accompanied with complex lesions such as extensive abscess formation is not recommended in most cases. Therefore, non-operative management followed by interval appendectomy (IA) for AA has been tried. Herein, we present three AA cases with specific etiology that underwent interval appendectomy.

**Case presentation:**

*Case 1*: A 68-year-old man was diagnosed AA with intestinal malrotation and intra-abdominal abscesses. He initially treated with conservative therapy and underwent laparoscopic IA after detailed preoperative examination.

*Case 2*: A 22-year-old man had been under treatment for pancolitis-type ulcerative colitis (UC), also bothered by right-lower abdominal pain several times a year. The appendix always appeared swollen on every CT taken during symptoms. He underwent laparoscopic IA; pathological finding revealed typical UC histological features in the resected appendix. After the surgery, he never suffered from terrible right lower abdominal pain.

*Case 3*: A 69-year-old woman complaining a right lower abdominal pain had undergone CT examination, which revealed AA with appendiceal mass, irregular wall thickness of the cecum, and mediastinal and para-aortic lymph node swelling. The operation was carried out after conservative therapy. The pathological diagnosis revealed *BRAF* mutated colorectal carcinoma. She had received systematic chemotherapy after the surgery, and all metastatic lesions have completely disappeared.

**Conclusion:**

Interval appendectomy provided us with much clearer anatomical information and precise therapeutic strategies, avoiding technical and general operative complications, and also induced fast recovery and short length of hospital stay. Interval appendectomy is a reasonable procedure and could be recommended in case of AA with some different etiology.

## Background

Appendectomy for AA is considered the most common emergency surgeries, and prompt appendectomy has long been a standard treatment for AA [1–3]. Currently, laparoscopic appendectomy (LA) becomes the first therapeutic choice for AA [2]. LA is a safe and effective procedure for the treatment of simple appendicitis, and this approach is superior to open appendectomy (OA) in terms of postoperative wound infections, analgesia requirement, length of hospital stay, return to work, and overall recovery [4, 5].

Immediate appendectomy is technically demanding with distorted anatomy, adhesive loops of bowel, and difficulty to close the appendiceal stump because of the inflamed tissues [6]. Then, early LA for AA with such conditions may be converted to OA, ileocecal resection, or right hemicolectomy [7].

Therefore, nonoperative management followed by IA for AA has been tried in many hospitals. Initially, AA may be managed in an elective nonsurgical manner including intravenous antibiotics and selective percutaneous drainage and then carrying out operation (mostly LA) [8–11]. This management has been performed especially in pediatric patients [2]. However, the validity of IA is still controversial in adult patients [2, 12].

Herein, we present three cases of AA with specific etiology who eventually underwent interval appendectomy, discussing beneficial effects of interval appendectomy for AA with different etiology.

## Case presentation

### Case 1

**Fig. 1:**
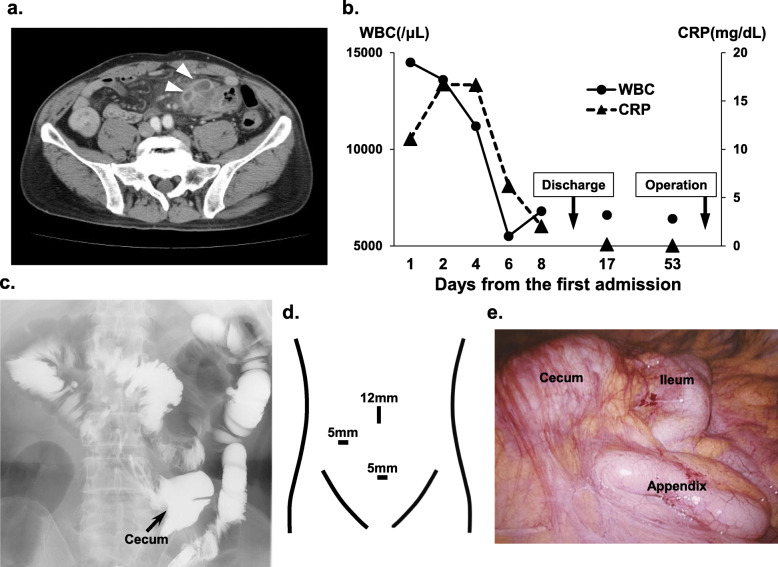
**a** CT findings: the cecum was located in the left lower side of the abdomen. The swollen appendix was surrounded by intra-abdominal abscesses (arrow heads). **b** The clinical course of this patient: he was discharged at day 9 and underwent an interval appendectomy at day 54. **c** The gastrografin enema findings: the cecum (arrow) located in left-lower side of the abdomen. **d** The trocar placement of the interval appendectomy: Note the mirror image trocar placement against an ordinal laparoscopic appendectomy. **e** Intraoperative findings: iliocecal structures were located in the left lower abdomen

A 68-year-old man complaining a left-lower abdominal pain had visited our hospital. CT revealed the cecum located in the left lower side of the abdomen and a swollen blind-end structure with intra-abdominal abscesses (Fig. 1a). Finally, he was diagnosed as AA with intestinal malrotation. He received conservative treatment with intravenous antibiotics, which ameliorated his symptom and inflammatory findings (Fig. 1b). He left the hospital at day 9. Afterwards, we carried out detailed preoperative examination on his outpatient visit. The gastrografin enema confirmed that the cecum was located in the left-lower side, revealing the presence of intestinal malrotation (Fig. 1c). We underwent a laparoscopic IA using a mirror-image trocar placement (Fig. 1d). The iliocecal structures were found at the left side of the abdomen (Fig. 1e). He was discharged from the hospital at the postoperative day 3.

### Case 2

**Fig. 2:**
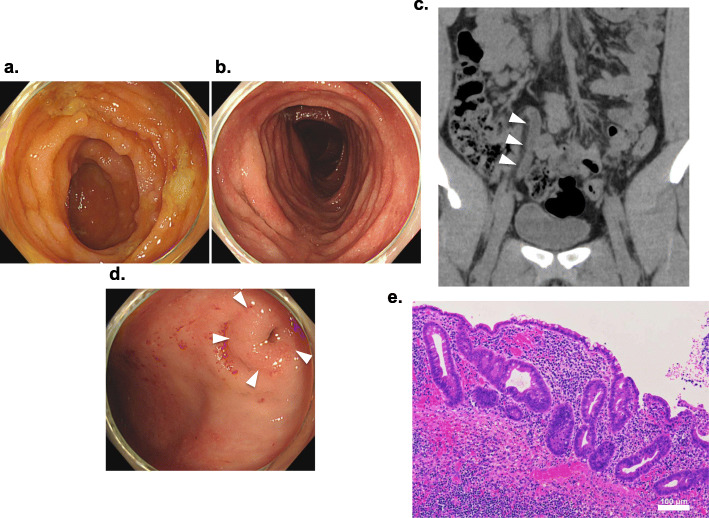
**a**, **b** Colonoscopic findings: continuous ulcerative lesions and erythema were observed. **c** CT fingings: CT revealed a swollen appendix on every symptomatic course (arrow heads). **d** Colonoscopic findings: The appendiceal orifice was almost normal (arrow heads). **e** Pathological examination: the pathological finding revealed inflammatory cells infiltration and crypt distortion in the resected appendix

A 22-year-old man had been under the treatment for pancolitis-type ulcerative colitis (UC) (Fig. 2a, b) and has been also bothered by right-lower abdominal pain several times a year. The appendix always appeared swollen on every CT taken during symptoms (Fig. 2c). On every onset of symptoms, he received intravenous followed by oral antibiotics, which always ameliorated his symptoms. Endoscopy examination revealed that the appendiceal orifice was almost normal (Fig. 2d). Both UC and AA might cause his right lower abdominal pain. He was treated with conservative treatment at first. He underwent a laparoscopic IA in order to obtain the accurate diagnosis. There were no operative complications, and he left the hospital 2 days after the operation. The pathological findings showed not only AA features (inflammatory cell infiltration) but also histological characteristics typical of UC (crypt distortion) in the resected appendix (Fig. 2e).

### Case 3

**Fig. 3:**
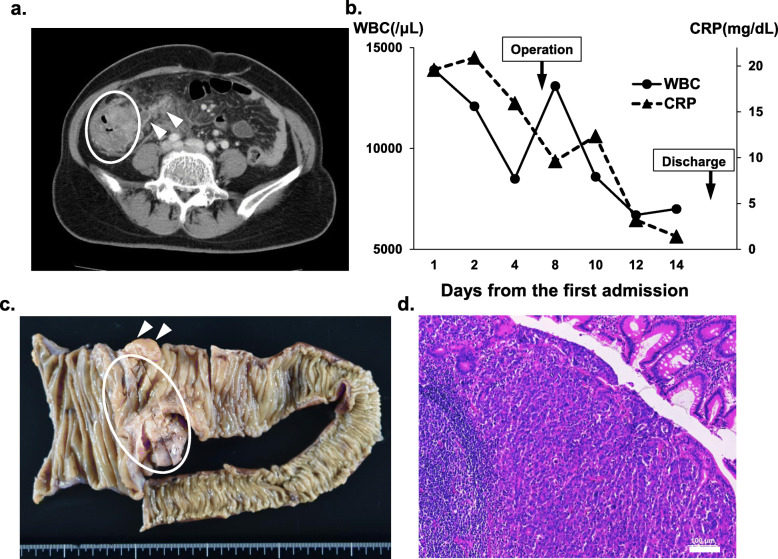
**a** CT findings: CT revealed acute appendicitis (arrow heads) with abdominal abscess and irregular wall thickness of the cecum (circle). **b** The clinical course of this patient: she underwent interval operation at day 7 and left the hospital at postoperative day 9. **c** A macro-finding of the resected organ: the arrow heads indicate the appendix. The oval indicates the tumor lesion. **d** Pathological examination: pathological features revealed poorly differentiated adenocarcinoma

A 69-year-old woman complaining a right lower abdominal pain undergone CT examination, which revealed ruptured AA with appendiceal mass and irregular wall thickness of the cecum (Fig. 3a), which made us suspect colon cancer. Moreover, there were mediastinal and para-aortic lymph node swelling, suspecting malignant lymphoma or lymph node metastases. Serum soluble IL-2R, CEA, and CA19-9 were 1298 U/ml (normal range; 122–496), 11.8 ng/ml (normal range; 0.1–5.0), and < 2 U/ml (normal range; < 37), respectively, and those serum findings made us suspect malignant lymphoma or epithelial neoplasm. Conservative therapy with intravenous antibiotics for AA was started. Seven days after the initiation of conservative therapy, her general condition and inflammatory signs were significantly improved (Fig. 3b). She underwent ileocolectomy (i.e., extended appendectomy because there were strong suspicions of malignancy) for making a pathological diagnosis. She left the hospital 9 days after the operation without any complications (Fig. 3b). The pathological features concluded poorly differentiated adenocarcinoma with peripheral lymph node metastases (Fig. 3c, d). The genotype analyses revealed right-side colorectal carcinoma with *BRAF* mutation, and liver metastasis lesion appeared later. She received chemotherapy (bevacizumab, 5-FU, folinate, oxaliplatin, CPT-11); 3 months later, either mediastinal and para-aortic lymph node swelling and all metastatic lesions had completely disappeared. She was kept on a complete response (CR) at the final visit (1 year and 2 months after the operation).

## Discussion

There are still controversies over the efficacy of IA for acute appendicitis [2, 12]. We presented here three interesting cases of AA who underwent IA which eventually proved to be a very effective therapeutic choice. So far, there have been case reports with a single case regarding the efficacy of IA. The current manuscript contains three cases with different unique etiologies. We can contrast one case with another in order to understand how the managements are different. Some reports suggested that the recurrence rate of AA during the waiting time for IA is 6–37%. And the complication rate of surgery for recurrent AA is as high as 3–23% [13]. However, especially in a phlegmon or appendiceal mass, IA may have some advantages, for example, providing a definite diagnosis, ruling out any underlying malignancy and avoiding unwanted injury to the surrounding tissue [9, 11, 14]. And also, the advantage of IA is to perform the operation at a time when the peritonitis has resolved, potentially resulting in fewer intraoperative and/or postoperative complications [15]. There are some analyses about cost-effectiveness of IA [16, 17]. However, cost-benefits of IA remain controversial. IA requires one more additional admission—one for conservative treatment and one for surgical therapy. IA might require much more medical resources. Nevertheless, IA provides a lot of benefits (accurate preoperative information, avoiding technical and general operative complications) and improves patient’s QOL (fast recovery and short length of postoperative hospital stay). Particularly, for cases with some characteristic etiology like we presented in the current manuscript, interval operation also provides us with precise therapeutic strategies that lead to well results and prognosis. Therefore, we advocate interval operation for AA with unique etiologies.

In case 1, AA with appendiceal mass was initially treated with conservative therapy, which enabled further examinations for intestinal malrotation. The anatomical information was extremely useful, making the operation safe and avoiding technical complications. IA seemed to induce his fast recovery and short length of hospital stay. Those results seemed to be one of the greatest benefits of IA.

Recent several reports have shown a significant negative association between appendectomy and UC [18–20]. However, the majority of the studies deal with the history of appendectomy before the development of UC [19, 20]. There are a few published or unpublished data about the course of UC when appendectomy is performed after UC diagnosis. Those reports suggested that the disease seems to become milder after appendectomy [21, 22]. Case 2 underwent IA electively, after which he never suffered from terrible right lower abdominal pain although there were no direct objective testimonies to the symptomatic improvements such as blood tests and pathological findings. In this case, IA seemed to ameliorate his UC condition.

There have been considerable studies on appendiceal adenocarcinoma. In a recent report about primary appendiceal carcinomas with an average age of 64 years old, 72% were T3/T4 tumors and 36.4% of them had lymph node metastasis. More than 20% were poorly differentiated, stage IV disease represented 23.2% of the cases, and 5-year overall survival was 47.5% for all stages [23, 24]. Such information reinforces the indication of any surgical intervention (appendectomy/ileocolectomy) required to treat appendiceal inflammatory mass after conservative treatment. In patients with metastatic disease, they often have peritoneum involvement. Patients who were submitted to surgical cytoreduction had a median recurrence-free survival of 1.2 years, and an overall survival of 4.2 years was achieved when patients could undergo a complete cytoreduction. Unfortunately, complete cytoreduction was achieved only in 21% of the patients [25]. In case 3, T3 tumor was accompanied with peripheral and distant lymph nodes, liver metastasis. We had undergone a complete cytoreduction after the conservative therapy for AA and were able to obtain the accurate diagnosis without any postoperative complications. Furthermore, in spite of the poor prognosis with *BRAF* gene mutation, those therapeutic strategies enabled the induction of precise early systematic chemotherapy, resulting in a well prognosis.

The ideal interval is thought to be approximately 2–3 months [26]. Needless to say, appendectomy can be performed easily once the inflammation abates. The interval of case 1 was ideal in this regard. However, the intervals of case 2 and case 3 were short. In case 2, he had repeatedly suffered abdominal pains caused by appendicitis which were always alleviated by a short course of antibiotics. There were no abscesses around the appendix. When an appendectomy may be performed easily, such short interval can be acceptable. In case 3, there were strong suspicions of malignancy and the appendix was perforated. Therefore, we performed the ileocolectomy as soon as the inflammation around the appendix somewhat abated in order to promptly start appropriate therapy against malignancy.

Although the beneficial role of IA for AA is still controversial, there are some advantages for selected AA cases with specific etiology. We present representative three AA cases with intestinal malrotation, inflammatory bowel disease and colorectal malignancy. In these cases, interval appendectomy provided us with much clearer anatomical information and precise therapeutic strategies, avoiding technical and general operative complications. Moreover, interval appendectomy also induced postoperative fast recovery and short length of hospital stay.

## Conclusion

Interval appendectomy is a reasonable procedure and could be recommended in case of acute appendicitis suspiciously having some different etiology.

## Data Availability

Data sharing is not applicable to this article, as no datasets were generated or analyzed during the current study.

## Abbreviations

AA: Acute appendicitis
IA: Interval appendectomy
UC: Ulcerative colitis
LA: Laparoscopic appendectomy
OA: Open appendectomy
CR: Complete response
